# Multi Sensor Fusion Framework for Indoor-Outdoor Localization of Limited Resource Mobile Robots

**DOI:** 10.3390/s131014133

**Published:** 2013-10-21

**Authors:** Leonardo Marín, Marina Vallés, Ángel Soriano, Ángel Valera, Pedro Albertos

**Affiliations:** Department of Systems Engineering and Control, Instituto Universitario de Automática e Informática Industrial, Universidad Politécnica de Valencia, Camino de Vera, E-46022 Valencia, Spain; E-Mails: mvalles@aii.upv.es (M.V.); ansovi@ai2.upv.es (Á.S.); giuprog@isa.upv.es (Á.V.); pedro@aii.upv.es (P.A.)

**Keywords:** mobile robots, pose estimation, sensor fusion, Kalman filtering, inertial sensors, robot sensing systems, dynamic model, embedded systems, global positioning systems, event based systems

## Abstract

This paper presents a sensor fusion framework that improves the localization of mobile robots with limited computational resources. It employs an event based Kalman Filter to combine the measurements of a global sensor and an inertial measurement unit (IMU) on an event based schedule, using fewer resources (execution time and bandwidth) but with similar performance when compared to the traditional methods. The event is defined to reflect the necessity of the global information, when the estimation error covariance exceeds a predefined limit. The proposed experimental platforms are based on the LEGO Mindstorm NXT, and consist of a differential wheel mobile robot navigating indoors with a zenithal camera as global sensor, and an Ackermann steering mobile robot navigating outdoors with a SBG Systems GPS accessed through an IGEP board that also serves as datalogger. The IMU in both robots is built using the NXT motor encoders along with one gyroscope, one compass and two accelerometers from Hitecnic, placed according to a particle based dynamic model of the robots. The tests performed reflect the correct performance and low execution time of the proposed framework. The robustness and stability is observed during a long walk test in both indoors and outdoors environments.

## 1. Introduction

Mobile robots are designed and built to perform several task simultaneously, they usually must communicate with neighbors to coordinate movements, navigate in the environment following a path, avoid obstacles, advance toward a goal point, *etc.* Most of these duties could not be performed without a precise knowledge of the robot current position and heading (*pose*) in a global reference frame [1,2]. This process, known as robot localization, is a challenging issue since the pose information is obtained from sensors subject to noise. If this fact is not taken into account, it could lead to great uncertainty while performing the localization process.

In order to improve the accuracy of the pose estimation, the several sensors available on the robot that measure the variables associated with the motion (acceleration, velocity, rotation, *etc.*) are used to localize the robot. These different measurements are combined by an algorithm that takes into account the different accuracy and noise levels of each sensor. The most commonly used fusion technique is the Kalman Filter (KF) or one of its variants for nonlinear systems [Extended (EKF), Unscented (UKF), *etc.*], all of them widely studied in the literature [3–6].

There are several examples of KF based localization algorithms developed for different types of mobile robots and sensors. Many works present only IMU and encoder based methods without a global sensor. For example, simulation results are presented in [7] where the EKF was used to fuse the encoder data with data from a gyroscope and in [8], where a range finder is also used but with a Hybrid EKF.

Other examples employ some mid-size mobile robots, such as the Pioneer family [9] with the filter algorithms being executed in a computer outside the mobile robot. This increases the computational resources available but limit the robot mobility as it adds more weight to the robot; it also adds communication delays between the robot and the fusion processing unit. For example, in [10] a Fiber-Optic Gyroscope is calibrated and integrated with the robot odometry using an EKF. This method shows good performance if the calibration is performed carefully and if the EKF with a nine state model is used. The tests use a Pioneer AT with a laptop-pc above it. In a similar way, in [11] an EKF fuse the encoder measurements with the output of an IMU while taking into account the wheel slip condition. It shows good performance in the tested skid-steered mobile robot. Also in [12] an UKF combines the information of a laser range finder with an IMU in a Pioneer 2-DxE mobile robot.

Advanced examples employ a robot with powerful processing units. Complex models and fusion schemes can be used in these cases and be implemented onboard the mobile robot, with the advantage of no communication delays and a more lightweight implementation but increasing the platform economic cost. In [13] a multi-rate EKF algorithm is presented, it combines the inertial measurements of a three-axis gyroscope and accelerometer with an optical navigation sensor (laser mice type) with the advantage of non-slip measurement. The filter is implemented onboard the custom build Ackerman type mobile robot using a CPU module with high memory capabilities showing good performance in the mapping of a 3-D pipeline.

Localization algorithms must perform the fusion with a global sensor for large distance runs to avoid unbounded error growth. These sensors can consist of a GPS sensor or an indoor global sensor (such as a zenithal camera [14] or a radio-based sensors as seen in [15]) depending on the application. There are many examples; most of them use an Ackerman type robot or a normal car adapted with and IMU, a GPS and a laptop to perform the fusion algorithm (for a general review see [16]).

Works in indoor environments include [17] that shows the EKF fusion of an ultrasonic satellite (U-SAT) with an IMU and encoders. The tests are performed with a custom made Ackerman robot and with an external computer to perform the fusion. Also in [18] an EKF is used with a Hybrid Localization Algorithm to fuse several ultrasonic sensor readings with the encoders, with good results in the Pioneer P3-DX mobile robot using ultrasonic beacons attached to the ceiling and a laptop computer to perform the fusion. In [19], a zenithal camera is used as an indoor GPS to be fused with the encoder data. The tests are performed in a low cost platform, the LEGO RCX. Good performance is shown despite the limited resources robot. This work was extended to the NXT robot in [20] with several IMU sensors but without a global sensor, thus the estimation diverges in long distance tests. In [21], several Radio Frequency Identification tags (RFID) placed in known locations along the indoor environment are identified by the mobile robot (Pioneer P3-DX) and used as a global position sensor. The sensor fusion is done by an EKF and by a Kalman quantized filter, both showing an improvement over using only the IMU and requiring less computational time with similar performance than a Particle Filter.

For outdoor environments, a basic example is shown in [22], where a Rover Dune robot with dual frequency GPS receiver uses an adaptive KF with Fuzzy Logic to improve the pose estimation. An EKF is used in [23] where an all-terrain mobile robots with four-wheel differential-drive skid-steering, where the encoder based dead-reckoning is corrected using a magnetic compass and a differential GPS (DGPS). Good performance is shown as the fusion scheme performs better than using the encoders or the DGPS alone, and when it is used in a cooperative multirobot localization scheme. A car is used in [24] with a three axis IMU (accelerometers gyroscopes, magnetometers) with a GPS fused by an EKF executed in a notebook. Tests show adequate operation even during GPS outages. In a similar configuration, [25] employs an EKF aided with a Neural Network with proper operation even with momentary interruptions in the GPS readings. Finally, in [26], a numerical algorithm adapts the model structure used in a KF. Extensive tests show low errors during GPS outages and good performance.

The great majority of these examples employ an algorithm to perform the sensor fusion that is computationally expensive, especially in the cases of fusion of multiple measurements (as large matrix inversion and manipulation are needed in these KF). This prevents the implementation onboard the robot processor (except for a few cases such as [13,19,20], with the associate disadvantage of requiring an external computer to perform the method, producing an unwanted delay in the control due to the communication between the controller computer and the robot.

This establishes an important issue: the localization method precision is as important as the computational resources used and the algorithm response time. Complex Fusion schemes based in nonlinear models and filters (UKF, particle filters [27], *etc.*) will provide a very precise estimation but will also require longer execution times as they perform complex calculations (large matrix inversion, matrix square root, *etc.*). On the other hand, linear or linearized fusion schemes take less time to calculate, but if the model is highly nonlinear it will diverge quickly. Also, if the localization information is missed or delayed, the data required in the navigation control algorithm would not be available producing a miss timed control action that can lead the system to an unstable behavior. Furthermore, as the pose estimation is not the only task of the robot, not all the available resources can be assigned to the localization. This establishes a compromise between the complexity and the precision of the localization technique, especially when the robot is resource-limited.

Observing the reviewed works, many of them use a global sensor in a regular sample time basis, even if the pose estimation error (using the IMU and the encoders) is small. In this case, there is no need to use the global sensor so regularly. A better approach, to save computational resources, would be to use the global information only when the estimation error covariance is big enough (when it is greater than a predefined limit). This event based update can save process time, bandwidth and extend the battery life. Event based solutions have been previously tested with success in other areas such as multirobot cooperative control [28], multi-agent consensus [29,30] and multi-agent agreement protocols [31], with success. The event strategy produces an exchange of information among the group members (sensors, controllers, robots, *etc.*) only when the error (in the reference or disturbance) exceeds a given bound, reducing the communication and processor load.

Considering these factors, this paper presents a resource-efficient multi sensor fusion framework with similar performance when compared to more complex fusion schemes but with lower computational, economical and communication costs and easy implementation on limited resource mobile robots. The proposed method implements the event based fusion algorithms defining an event that depends not on the reference error (as the previous approaches) but in the covariance of the estimation error. Along with the fusion framework, two experimental low cost LEGO NXT based platforms are presented to indoor and outdoor navigation respectively.

After this review, the paper is organized as follows. In Section 2, the event based sensor fusion algorithms are presented along with the mathematical models needed by the KF equations. In Section 3, the multi sensor fusion framework implementation is described for each of the proposed experimental platforms. In Section 4 the performance and run time tests are presented for the indoor and outdoor scenarios. Finally, some conclusions are drafted in the last section.

## 2. Event Based Fusion Algorithms

The mathematical models and the proposed KF algorithms are exposed next.

### 2.1. Mobile Robots Models

#### 2.1.1. Differentially Driven Wheeled Robot

**Figure 1.**
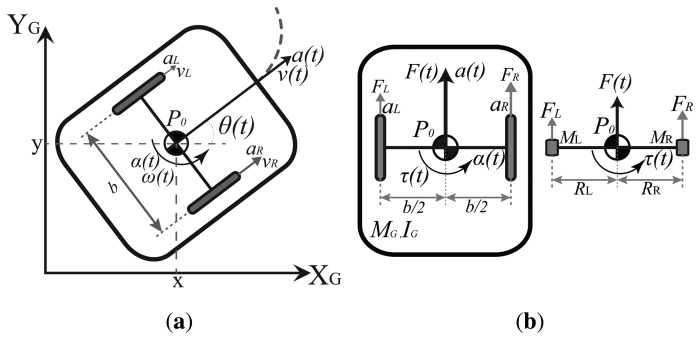
Differentially driven wheeled robot. (**a**) Kinematics; (**b**) Dynamics.

A differentially driven wheeled robot consists of a rigid body with mass *M_G_* and moment of inertia *I_G_* with two non-deformable non-orientable (fixed) wheels separated a distance *b* and moved by two motors that apply two linear forces *F_R_* and *F_L_* as shown in Figure 1a,b. The wheels are conventional and they satisfy the pure rolling without slipping condition [32]. For this work, the “Slow Speed Motion” condition can be assumed as the maximum velocity of the experimental platforms based on the LEGO NXT, is less than 5 *m*/*s*. With this condition, the wheels are assumed without slip [33]. Also, the movement of the robot is restricted to a horizontal plane and its center of mass is denoted by *P*_0_ that also corresponds to the center of gravity.

The kinematic model represents the robot velocities evolution in a fixed inertial frame. The robot pose is defined by its position ***P***_0_ = (*x*, *y*) with the heading angle *θ* in the *Global* reference frame (*X_G_*,*Y_G_*) in Figure 1a. By knowing the linear and angular velocities (*υ* and *ω*) in the *Local* frame (*X_L_*,*Y_L_*), the *global* velocities are defined as:

(1) $$\left[\begin{array}{c} \dot{x}\\ \dot{y}\\ \dot{\theta } \end{array}\right]=\left[\begin{array}{cc} \cos\theta & 0\\ \sin\theta & 0\\ 0 & 1 \end{array}\right]\;\left[\begin{array}{c} \upsilon \\ \omega \end{array}\right]$$

By discretizing and recursively integrating Equation (1) with sample time *T_s_*, the following robot *global* pose ***L*** is obtained:

(2) $$L_{k}=\left[\begin{array}{c} x_{k}\\ y_{k}\\ \theta_{k} \end{array}\right]=\left[\begin{array}{c} x_{k-1}\\ y_{k-1}\\ \theta_{k-1} \end{array}\right]+\left[\begin{array}{c} \upsilon_{k-1}T_{s}\cos(\theta_{k-1}+0.5T_{s}\omega_{k-1})\\ \upsilon_{k-1}T_{s}\sin(\theta_{k-1}+0.5T_{s}\omega_{k-1})\\ T_{s}\omega_{k-1} \end{array}\right]$$

The kinematic relation between *υ* and *ω* and the linear velocities in the wheels (*υ_L_*, *υ_R_*) is shown in:

(3) $$\left[\begin{array}{c} \upsilon \\ \omega \end{array}\right]=\left[\begin{array}{ll} 1/2 & 1/2\\ -1/b & 1/b \end{array}\right]\;\left[\begin{array}{c} \upsilon_{L}\\ \upsilon_{R} \end{array}\right]\Rightarrow \left[\begin{array}{c} a\\ \alpha \end{array}\right]=\left[\begin{array}{ll} 1/2 & 1/2\\ -1/b & 1/b \end{array}\right]\;\left[\begin{array}{c} {\dot{\upsilon }}_{L}\\ {\dot{\upsilon }}_{R} \end{array}\right]$$

By deriving this equation, a kinematic relation between linear and angular accelerations *a* and *α* and the accelerations at the robot wheels, is obtained. By using two 3D accelerometers, placed above each wheel, Equation (3) will give the robot accelerations that can be integrated to obtain the velocities that will be used as inputs in Equation (2). But a better approach is to take into account the robot dynamics to obtain the global accelerations.

The dynamic model represents the robot linear and angular accelerations (*a,α*) evolution in terms of the forces applied to it in the wheels (*F_L,R_*) and the angular moment (*τ*). The models in the literature represent the dynamics in the *global* reference frame (for example [34–36]) but these yield too complex nonlinear models with difficult implementation. Also, as no direct measurements of the motor forces or input currents are available in most robots, obtaining the dynamic model in terms of the linear accelerations is convenient. Instead of using directly the second Newton's Law in the center of mass, a better approach is to consider the robot rigid body as a dynamically equivalent particle system, formed by two mass particles *M_L_* and *M_R_* joined by a massless connector of constant length *b* = *2R_N_* (with *R_N_* = *R_L_* = *R_R_*) as shown in Figure 1b. Using this equivalence and the conditions stated by [37] (full development in Appendix A), the accelerations are obtained as:

(4) $$\begin{array}{l} a=\gamma \frac{M_{G}}{M_{G}}(a_{R}+a_{L})\Rightarrow a=\gamma (a_{R}+a_{L})\\ \alpha =\gamma \frac{M_{G}R_{N}}{I_{G}}(a_{R}-a_{L})\Rightarrow \alpha =\lambda \gamma (a_{R}-a_{L}) \end{array}$$

The parameters of the particle system (two constants *γ* and *λ* and the distance *R_N_*) are obtained for different robots shapes as shown in Table 1. For example, in case of a differential robot with rectangular shape (as the one proposed in the platforms description section), the parameters to be used are the ones referred as “solid box”.

By discretizing and recursively integrating the accelerations (integrating the simple kinematic model *a* = *υ̇*,*α* = *ω̇*) and substituting Equation (4), the robot *local* linear dynamical model is obtained in Equation (5).

(5) $$\left[\begin{array}{c} \upsilon_{k}\\ \omega_{k} \end{array}\right]=\left[\begin{array}{ll} 1 & 0\\ 0 & 1 \end{array}\right]\;\left[\begin{array}{c} \upsilon_{k-1}\\ \omega_{k-1} \end{array}\right]+\left[\begin{array}{ll} T_{s} & 0\\ 0 & T_{s} \end{array}\right]\;\left[\begin{array}{c} a_{k-1}\\ \alpha_{k-1} \end{array}\right]$$

By substituting Equation (5) as inputs of Equation (2) the robot *global* nonlinear dynamical model is written as:

(6) $$\left[\begin{array}{l} x_{k}\\ y_{k}\\ \theta_{k}\\ \upsilon_{k}\\ \omega_{k} \end{array}\right]=\left[\begin{array}{l} x_{k-1}\\ y_{k-1}\\ \theta_{k-1}\\ \upsilon_{k-1}\\ \omega_{k-1} \end{array}\right]+\left[\begin{array}{c} \upsilon_{k-1}T_{s}\cos(\theta_{k-1}+0.5T_{s}\omega_{k-1})\\ \upsilon_{k-1}T_{s}\sin(\theta_{k-1}+0.5T_{s}\omega_{k-1})\\ T_{s}\omega_{k-1}\\ T_{s}a_{k-1}\\ T_{s}\alpha_{k-1} \end{array}\right]$$

The models (4)-(6) are used in the event based algorithms for the differential indoors platform.

#### 2.1.2. Ackermann Steering Mobile Robot

**Figure 2.**
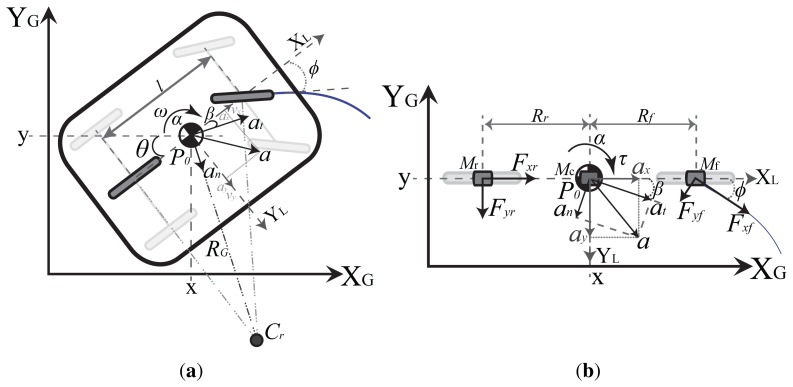
Ackermann steering mobile robot. (**a**) Kinematics; (**b**) Dynamics.

An Ackermann steering robot consists of a rigid body with center of mass and gravity denoted by *P_0_*, turning radius *R_G_*, mass *M_G_* and moment of inertia *I_G_*, with two non-orientable (fixed) rear wheels separated a distance *b* between them and a distance *l* from two orientable front wheels (also with a distance *b* between them) as shown in Figure 2a. The Ackermann steering system modifies the heading of the front wheels in a way that, at low speeds, all the tires are in pure rolling without lateral sliding [38]. This is accomplished when each wheel follows a curved path with different radius but with one common turn center *C_r_*, as shows Figure 2a. This system is analyzed using the bicycle model [33,38] assuming low speed planar motion and again, the non-slip condition (*υ_y_* ≈ 0). By this, the mean of the inside and outside front wheels steer angles (*ϕ_i_* and *ϕ_o_*) is used (*ϕ*) and the back wheels are considered as one single wheel where the motor force is applied (Figure 2b).

Following the previous analysis for the differential case, the models (5) and (6) can be also used for the Ackermann robot by adjusting the dynamic model to form a three mass system with particles *M_r_*, *M_c_* and *M_f_* joined by two massless connectors of constant length *R_r_* and *R_f_* (with *R_f_* = *R_r_* = 0.5*l*) as shown in Figure 2b with forces applied in the front (*F_f_*) and rear (*F_r_*) equivalent wheels. Once again, following [37] and a similar development as in Appendix A, the accelerations are obtained as:

(7) $$\begin{array}{l} a=a_{x}=\lambda_{a}(a_{f,x}+a_{r,x})\\ \alpha =\lambda_{\alpha }\gamma (a_{f,y}-a_{r,y}) \end{array}$$

The parameters of the particle system for the Ackermann robot with rectangular form, with length *c* and width *b* are obtained as:

(8) $$\begin{array}{ll} \gamma =\frac{1}{6}\left(1+{\left(b/c\right)}^{2}\right), & \lambda_{a}=0.5\\ \lambda_{\alpha }=\frac{6}{c\left(1+{\left(b/c\right)}^{2}\right)}, & \lambda_{\alpha }\gamma =\frac{1}{c} \end{array}$$

These parameters in Equation (8) are the ones used in case of an Ackermann robot with a rectangular shape (as the one proposed in the platforms description section). Finally, the kinematic relation between *ϕ* and *ω* is shown in:

(9) $$\omega =\left(\upsilon_{x}\tan\phi \right)/l$$

The models in Equations (5)–(7), are used in the event based algorithms for the Ackermann outdoors platform.

### 2.2. Event Based Fusion Algorithms

In this section the proposed event based fusion framework is described, but first a short review of the time based method is presented. To simplify the notation, the models used in the algorithms are referred as *linear* when it can be written as Equation (10) with input ***u****_k_* ∈ ℜ*^u^*, measurement ***z****_k_* ∈ ℜ*^u^* and state ***x****_k_* ∈ ℜ*^u^*, or *nonlinear* when a non-linear function ***f*** is needed to relate the state at time *k* with the previous time step *k* − 1 and ***h*** to relate the *x_k_* to *z_k_* as Equation (11) shows. Also, the terms ***w****_k_*, *υ_k_* represent the process and measurement noises at time *k* that have an independent, white probability distribution with zero mean.

(10) $$x_{k}=Ax_{k-1}+Bu_{k-1}+w_{k-1},\;z_{k}=Hx_{k}+\upsilon_{k}$$

(11) $$x_{k}=f(x_{k-1},u_{k-1},w_{k-1}),\;z_{k}=h(x_{k},\upsilon_{k})$$

#### 2.2.1. Time Based Fusion Algorithm

This method is the “traditional” one, which uses the model in Equation (6) and all the available measurements to estimate the robot pose at every time *k*. For example, in the differential case, if the available measurements are *υ_enc_* and *ω_enc_* from the encoders, *ω_gyr_* from a gyroscope, *ω_comp_* from a compass, *a_L_*, *a_R_* from two 3D accelerometers [placed as shown in Figure 1a and used in Equation (3)] and the global pose information *L_GM_* = (*x*, *y*, *θ*)*_GM_*, obtained from a zenithal camera, then ***z**_k_* = [*x_GM_ y_GM_ θ_GM_ υ_enc_ ω_enc_ ω_gyr_ ω_comp_*]*^T^* is used with ***u**_k_* = [*a_L_ a_R_*]*^T^* in Equation (6). In a similar way, if *L_GPS_* = (*x*, *y*, *θ*)*_GPS_* is the global pose information obtained from a GPS then, the variables used in Equation (6) for the Ackermann case are ***z**_k_* = [*x_GPS_ y_GPS_ θ_GPS_ υ_x,enc_ ω_enc_ ω_gyr_ ω_comp_*]*^T^* and ***u**_k_* = [*a_R_ a_F_*]*^T^* from two 3D accelerometers [placed as shown in Figure 2b and used in Equation (7)].

The model (7) is used in the EKF algorithm (Algorithm 1) which performs the sensor fusion at every time *k* (time based) using the process and measurement noises covariance matrices ***Q**_k_* and ***R**_k_* respectively and obtains the state vector estimate ***x**^_k_ i.e.*, the robot pose in Equation (6) and the covariance of the estimation error ***P**_k_*. This method has all the available information at any time *k* (all available sensors are used in *z_k_* and *u_k_*) but requires large matrix inversion to obtain the filter gain *K_k_*, thus large memory and resources are needed. The UKF [5] can also be used instead of the EKF using the same *x_k_*, *u_k_* and *z_k_* if the robot has enough resources to implement it.



**Algorithm 1**: Recursive EKF algorithm
 **Input** : *u_k_*,*z_k_*,*x_k_*_−1_,*P_k_*_−1_ **Output**: *x̂_k_*,*P_k_* **Data**: *f* and *h* from model (6), *Q_k_,R_k_* Initialization: *x_0_*,*P_0_* **for**
*current time k*
**do**  Prediction Step:  *x̂* = *f* (*x̂_k_*_−1_, *û_k_*_−1_, 0)  
$A_{k}=\frac{\partial f}{\partial x}|{\hat{\text{X}}}_{k-1},u_{k-1},0$  
$H_{k}=\frac{\partial h}{\partial x}|{\hat{\text{X}}}_{k},0$  
$W_{k}=\frac{\partial f}{\partial w}|{\hat{\text{X}}}_{k-1},u_{k-1},0$  
$V_{k}=\frac{\partial h}{\partial \upsilon }|{\hat{\text{X}}}_{k},0$  
$P_{k}=A_{k}P_{k-1}A_{k}^{T}+W_{k}Q_{k-1}W_{k}^{T}$  Correction Step:  
$K_{k}=P_{k}H_{k}^{T}{\left(H_{k}P_{k}H_{k}^{T}+V_{k}R_{k}V_{k}^{T}\right)}^{-1}$  *x̂* = *x̂_k_* + *K_k_* [*z_k_* − *h*(*x̂_k_*, 0)]  *P_k_* = (I − *K_k_ H_k_*) *P_k_* **end**


#### 2.2.2. Event Based Fusion Algorithm

The event based approach fuse the sensor information when an event passes a predefined limit. In order to adapt the event scheme to the localization problem *z_k_* is divided into the local and global measurements. By this, using as example the variables from in the differential case, the robot pose is determined every time *k* using Equation (6) in the EKF and the local ***z**_k_* = [*υ_enc_ ω_enc_ ω_gyr_ ω_comp_*]*^T^* with *u_k_*= [*a_L_ a_R_*] used in Equation (4), and using the global pose *L_GM_* to correct the local estimation in the event based scheme. The event in this paper is not defined based on the reference errors, but in the estimation error covariance (pose section of *P_k_* = *P_kp_* in the EKF), that is a clear indicator of the “health” of the localization solution. By this, the event approach will use *L_GM_* only when the error in the estimation is big enough. The event can be defined in multiple ways using ***P****_k,xy_*. For example, a ratio ***R****_A_* of the robot area *A_NXT_* and the 3 – *σ* error interval ellipsoids *area A_ellip_* can be used, as defined in Equation (12), where the ellipsoids axis are *a_σ_* and *b_σ_* and *l* = 3 for the 3 – *σ* error. In this event definition, when ***R****_A_* exceeds a certain level ***R****_A,lim_*, e.g., 1.6 (indicating that the error area is 1.6 times the robot area) then *L_GM_* is used to correct the robot pose. This limit is chosen as a compromise between the number of calls (energy and processor time consumption) and the desired precision of the estimated pose, being a reasonable range 0.125 ≤ ***R**_A,lim_* ≤ 2 for the LEGO NXT (details on this *R_A,lim_* threshold are given in Section 4.2).

(12) $$\begin{array}{ll} P_{k,xy=}\left[\begin{array}{ll} \sigma_{x}^{2} & \sigma_{xy}^{2}\\ \sigma_{xy}^{2} & \sigma_{y}^{2} \end{array}\right] & a_{\sigma }=\sqrt{\frac{2l^{2}\left|P_{xy}\right|}{\sigma_{x}^{2}+\sigma_{y}^{2}+T\sigma }}\\ T_{\sigma }=\sqrt{\sigma_{x}^{4}+\sigma_{y}^{4}-2\sigma_{x}^{2}\sigma_{y}^{2}+4\sigma_{xy}^{4}}, & b_{\sigma }=\sqrt{\frac{2l^{2}\left|P_{xy}\right|}{\sigma_{x}^{2}+\sigma_{y}^{2}-T\sigma }}\\ A_{NXT}=b\cdot c & A_{\text{ellip}}=\pi a_{\sigma }b_{\sigma } \end{array}$$

To correct the EKF pose *x_k,p_* with *L_GM_*, in the differential case (Algorithm 2), the difference (Δ*X*,Δ*Y*) is added to the EKF position output (*x*,*y*)*_EKF_* as stated in Equation (13). This is obtained as the difference between the estimated EKF position at the time of the event *x̂_eEKF_*, *ŷ_eEKF_* and the measured (*x*, *y*) values in *L_GM_*; compensating any communication delay Δ*T* (assumed known or measurable) using the velocities in the global axis *V x_e_, V y_e_* stored in the robot memory also at the event time (details on the delay measurement are given in the platform description). The heading *θ̂_EKF_* is replaced by the measured value in *L_GM_* and *P_kp_* = *P_k,x,y,θ_* is replaced by the corresponding values of *P_0_* which resets the event generator. To preserve the linear displacement assumed by Equation (13), the global updates are not performed when the robot is in hard motion (i.e. when applying a big control action to the motors to negotiate a curve or to avoid an obstacle). This is done checking the reference error in the wheels velocities, if it exceed a certain value (|*e_L,R_*| *>* 2) then the robot is assumed to be in a hard curve. In this case, the algorithm waits one second and then checks again this condition. If it is false, then it makes again the global update procedure and outputs the corrected pose (details on this *e_L,R_* error threshold and the waiting time are given in Section 4.2).



**Algorithm 2**: Event based KF, Differential case
**Input** : *u_k_*,*z_k_*,*x_k_*_−1_,*P_k_*_−1_,*L_GM_***Output**: *x̂_k_*,*P_k_***Data**: A, B and H from model (5), *Q_k_*,*R_k_*,*A_NXT_*,*Q_x_*Initialization: *x*_0_,*P*_0_**for**
*current time k*
**do** Prediction Step KF: *x̂_k_* = A*x̂_k_*_−1_+*Bu_k_*_−1_ *P_k_* = *AP_k_*_−1_*A^T^*+*Q_k_* Correction Step KF: 
$K_{k}=P_{k}H_{k}^{T}{\left(H_{k}P_{k}H_{k}^{T}+R_{k}\right)}^{-1}$ *x̂_k_* = *x̂_k_*+*K_k_*[*z_k_*−*Hx̂_k_*] *P_k_* = (*I*−*K_k_ H_k_*)*P_k_* Pose estimation using (2) Covariance propagation using (14) 3 − *σ* ellipsoid area *A_ellip_* using (12) ***R**_A_* = *A_ellip_*/*A_NXT_* **if *R****_A_* > *R_A,lim_*
**then**  Global Correction Step:  (same as Algorithm 4) **end****end**

(13) $$\begin{array}{c} \Delta X=({\hat{x}}_{e,EKF}+Vx_{e}\Delta T)-x_{GM}\\ \Delta Y=({\hat{y}}_{e,EKF}+Vy_{e}\Delta T)-y_{GM} \end{array},\begin{array}{c} x=x_{KF}+\Delta X\\ y=y_{KF}+\Delta Y\\ \theta =\theta_{GM} \end{array}$$

Another approach to correct the EKF pose is used in the Ackermann case (Algorithm 3) that takes into account the global sensor accuracy. This is done by using the EKF output (pose states and error covariances) as the prediction step of a linear KF. The model used in the fusion is Equation (10) with *A* and *H_p_* both being the identity matrix 
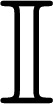
_3,3_ and no input. The *R_k,GM_* values are obtained from the global sensor accuracy. With this, the KF will fuse the local estimate with *L_GPS_*. The resulting *P_k,p_* decreases when the pose is corrected, resetting the event. The event based EKF is shown in Algorithm 4 with the correction of the differential case.



**Algorithm 3:** Event based KF, Ackermann case
**Input** : *u_k_*,*z_k_*,*x_k_*_−1_,*P_k_*_−1_,*L_GPS_***Output**: *x̂_k_,P_k_***Data:** A, B and H from model (5), *Q_k_*,*R_k_*,*A_NXT_*,*Q_x_*,*H_p_*,*R_k,GM_*,*P_k,p_*Initialization: *x*_0_,*P*_0_**for**
*current time k*
**do** Prediction Step KF: *x̂_k_* = *Ax̂_k_*_−1_+*Bu_k_*_−1_ *P_k_* = *AP_k_*_−1_*A^T^*+*Q_k_* Correction Step KF: 
$K_{k}=P_{k}H_{k}^{T}{\left(H_{k}P_{k}H_{k}^{T}+R_{k}\right)}^{-1}$ *x̂_k_* = *x̂_k_* + *K_k_*[*z_k_*−*Hx̂_k_*] *P_k_* = (*I*−*K_k_ H_K_*)*P_k_* Pose estimation using (2) Covariance propagation using (14) 3 −*σ* ellipsoid area *A_ellip_* using (12) ***R****_A_* = *A_ellip_*/*A_NXT_* **if *R***_*A*_ > *R_A_,_lim_*
***And N****_sat_* > *N_sat,min_*
**then**  Global Correction Step, pose KF  
$K_{k,KF}=P_{k,p}H_{p}^{T}{\left(H_{p}P_{k,p}H_{p}^{T}+R_{k,GM}\right)}^{-1}$  *x̂_k,p_* = *x̂_k_*_−1_,*_p_* +*K_k,KF_*(*L_GPS_*−*H_p_ x̂_k_*_−1_,*_p_*)  *P_k_*,*_p_* = (*I*−*K_k_,_KF_ H*) *P_k,p_* **end****end**


**Algorithm 4:** Recursive EKF algorithm with event based update
**Input:**
*u_k_*,*z_k_*,*x_k_*_−1_,*P_k_*_−1_,*L_GM_***Output:**
*x̂_k_,P_k_***Data:***f* and *h* from model (6), *Q_k_,R_k_,A_NXT_*Initialization: *x*_0_,*P*_0_**for**
*current time k*
**do** Prediction Step EKF: (same as Algorithm 1) Correction Step EKF: 
$K_{k}=P_{k}H_{k}^{T}{\left(H_{k}P_{k}H_{k}^{T}+V_{k}R_{k}V_{k}^{T}\right)}^{-1}$ *x̂_k_* = *x̂_k_*+*K_k_*[*z_k_*−*h*(*x̂_k_*,0)] *P_k_* = (*I*−*K_k_ H_k_*) *P_k_* 3 − *σ* ellipsoid area *A_ellip_* ***R****_A_* = *A_ellip_*/*A_NXT_* using (12) **if *R****_A_* > *R_A,lim_*
**then**  Global Correction Step:  *x_EKF_* = *x_EKF_* + Δ*X*  *y_EKF_* = *y_EKF_* + Δ*Y*  *θ_EKF_* = *θ_GM_*  *P_k,p_* = *P_0,x,y,θ_* **end****end**


The event based algorithm is adjusted to allow its implementation in resource limited robots and also adapted to the sensors available according to the navigation environment as described next, for the indoor differential case and the outdoor Ackermann case.

#### 2.2.3. Event Based KF, Differential Case

In the differential robot, instead of using Equation (6) with the full state, the velocities in Equation (5) are used to perform the local fusion, and then, Equation (2) is used to obtain the robot pose. As Equation (5) is linear, the fusion is performed using the KF instead, saving more computational resources (compared to Algorithm 1 and 4) as the linearization step of the EKF is not needed. By this, *x_k_*=[*υ*, *ω*], ***z**_k_*=[*υ_enc_ ω_enc_ ω_gyr_ ω_comp_*]*^T^* and again ***u**_k_*=[*a_L_ a_R_*]*^T^* is used in Equation (4). Also *L_GM_* is used to correct the local estimation in the event based scheme. As the uncertainty in the velocity measurement is not propagated to the pose estimation as does the previous methods, a linear recursive approximation is used to propagate the covariance from the velocity ***P**_k,υ,ω_* to the pose ***P**_k,p_* and also propagate it in time [6,39] as shows in Equation (14). In this, ∇***F**_u_* is the gradient operator applied to Equation (2) respect to the inputs (*υ_x_*,*ω*) and ***Q****_x_* are set equal to the *Q_k_*, *pose* terms in the EKF. The fusion filter is shown in Algorithm 2.

(14) $$P_{k,p}=P_{k-1,p}+\nabla F_{u}P_{k,\upsilon_{x},\upsilon_{y},\omega }\nabla F_{u}^{T}+Q_{x}$$

#### 2.2.4. Event Based KF, Ackermann Case

For the Ackermann robot, the same algorithm of the differential case is employed but with *x_k_* = [*υ_x_*, *ω*] and ***z**_k_* = [*υ_x,enc_ ω_enc_ ω_gyr_ ω_comp_*]*^T^*. Once again, ***u**_k_* = [*a_R_ a_F_*]*^T^* is used in Equation (7) as inputs of Equation (5) to perform the local fusion and then obtain the robot pose with Equation (2) that is corrected with *L_GPS_*. The event used is a combination of ***R****_A_* with and additional requirement for the satellites available *N_sat_* in the GPS sensor. A minimum *N_sat,min_* is defined to improve the accuracy of *L_GPS_* and avoid a correction of the local estimate with an inaccurate measurement. Also, the *R_k,GM_* values in the KF are increased (if *N_sat_* is low) or decreased (if *N_sat_* is high) online according to the current *N_sat_*, with this, *L_GPS_* measurements are taken more in account in the KF fusion when there are more satellites used in the GPS sensor. The fusion filter is shown in Algorithm 3.

As the proposed algorithms (Algorithm 4, 2 and 3) have a reduced *z_k_* and *x_k_* (comparing to the time based scheme Algorithm 1), the matrix inversion required for *K_k_* is performed faster, reducing the memory use and leaving resources for other tasks. The proposed experimental platforms are presented next.

## 3. Platforms Description

Two experimental low cost platforms are developed using the LEGO ®NXT mobile robot platform due its low cost and easy setup. Its control unit is the NXT based on an ARM7 32-bits microcontroller with 256 Kbytes FLASH and 64 Kbytes of RAM. It also has an USB 2.0 port and a wireless Bluetooth class II, V2.0 device with 4 inputs and 3 analog outputs. If offers basic sensors such as touch, light, sound and distance, but it also can work with third party sensors like vision cameras, magnetic compass, accelerometers, gyroscopes, *etc.* [40,41]. The actuators are DC motors with integrated encoders sensors of 1-degree resolution [42]. The programing platform used is the Java based LeJOS, due to the programming advantages in the communications between a robot and a supervising PC. The main limitation of this robot is the memory being only able to manage up to 255 local variables, 1024 constants, 1024 static fields and a maximum code length of 64 *kb* when programing with LeJOS. Because of this, it is an excellent option to implement the proposed filters as it does not have enough memory to implement a large KF with many states and measurements.

All the local sensors are calibrated to remove any bias present, and their values are set to the S.I. system. Also for the encoders, the calibration procedure exposed in [2] is followed to improve the measurements accuracy. The preprocessing is done to obtain *υ* and *ω* from the sensor data by integrating the encoders reading with a sample time of *T_s_* = 50*ms* to obtain the wheels velocities employed in Equation (3) to obtain *υ_enc_* and *ω_enc_*. The gyroscope measures directly *ω_gyr_*. And *θ_comp_* is measured from the compass in a range [0, 2*π*], its change every *T_s_* is used to obtain *ω_comp_*. As both, the calibration and the preprocessing steps, depend on the used sensors and robot, these processes are performed for every robot used in the experiments and also when a sensor or a motor is replaced. The experimental platforms are described next.

### 3.1. Differential Indoors LEGO NXT

**Figure 3.**
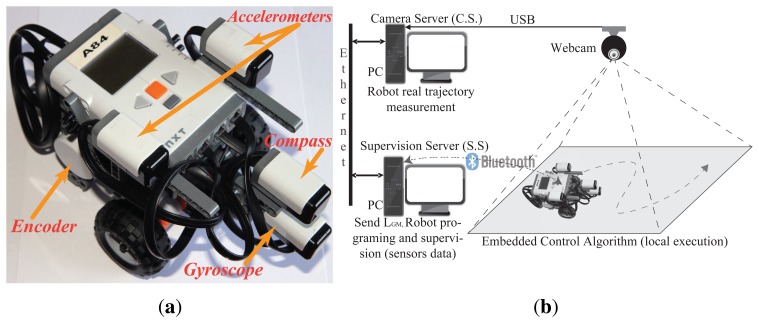
Differential LEGO NXT mobile robot with indoor zenithal camera sensor. (**a**) Differential Platform; (**b**) Indoor Global Sensor Setup.

The Differential LEGO NXT robot used in the tests is shown in Figure 3a. The sensors used in the fusion scheme are two accelerometers placed above the center of each wheel axis (for the dynamic model, Figure 1b), one gyroscope, one magnetic compass and the wheels encoders as marked in Figure 3a.

The global sensor scheme is the system shown in Figure 3b that is composed by the camera (640 × 480, 30 fps), the server that processes the image (camera server—C.S.) and the server that communicates with the robot (supervision server—S.S.). The C.S. executes a Java based program that constantly gets the image from the webcam, and makes the image processing to obtain the global measured pose ***L****_GM_* along with the time that the server took to obtain this value *T_cam_* (50 ms in average but the sent value is the actual measured time). This information forms the message that is communicated to the S.S. This server also stores every measure in a file and a video with the camera capture for the later analysis of the robot motion.

The delay Δ*T* is assumed known or measurable in the proposed algorithms. This can be obtained as a mean value from several experiments and this constant value can be used directly in the algorithms as a numeric approximation of the real delay time. But a better approach is to use the real measurement values, obtained each time the global information is accessed. This way, no approximation or mean value of Δ*T* is used in the algorithm, as the actual value is obtained by the following method. When the event is generated, the robot asks the S.S. via Bluetooth for a global measurement and the output provided is the EKF/KF estimation until the message from the S.S. arrives. As the S.S. executes a Java program that is continuously listening for the robot calls, the request is processed immediately, so it sends the last message from the C.S that contains ***L****_GM_* and *T_cam_* to the robot. When this message is received, the robot has also measured the round trip time *T_ss_* through an internal timer. As the measurement of *T_ss_* also includes the time of the image processing ***T****_cam_*, the communication delay is obtained as Δ*T* = (*T_s__s_*−*T_cam_*) /2; given that ***L****_GM_* is obtained as soon as the message is received by the S.S. and considering that the trip times are equal (from the robot to the S.S. and vice versa). The delay is used in Equation (13) to correct the EKF pose (Algorithm 2), and as the update is restricted to not be performed during the hard movements, the heading angle *θ* is assumed to be constant during *T_ss_*.

It should be noted that Δ*T* is considered as an input to the event based algorithms and in the present paper is obtained using the real measurements of *T_ss_* and *T_cam_*. Other approaches can be used to obtain it but for the proposed platforms, the measurement equation of Δ*T* is considered sufficient.

### 3.2. Ackermann Outdoors LEGO NXT

**Figure 4.**
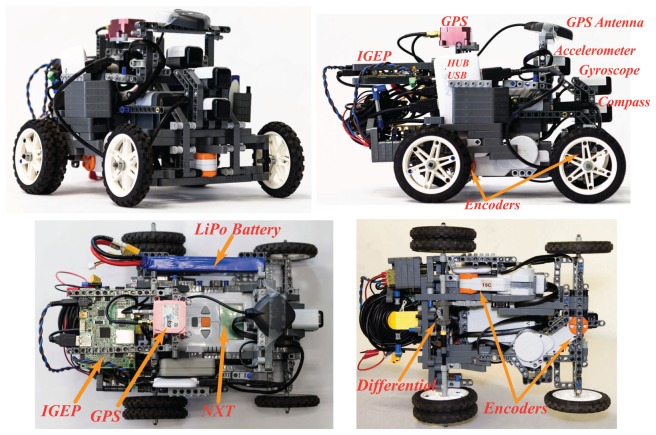
Ackermann LEGO NXT mobile robot with outdoor GPS sensor.

The Ackermann LEGO NXT robot used in the tests is shown in Figure 4. Once again, the sensors used in the fusion scheme are two accelerometers placed above the center of each wheel axis (for the dynamic model, Figure 2b, one gyroscope, one magnetic compass and the wheels encoders, these sensors are marked in Figure 4. Also, a differential is added to transmit the motor movement to the wheels while allowing them to rotate at different speeds (being *υ_x_* the average of them), allowing the correct turn of the vehicle.

As a global sensor, a SBG Systems IG-500N GPS [43] is used. It has an internal IMU that is not used during the test performed, only the GPS measurements along with *N_sat_* are employed to observe the behavior of the proposed algorithm with the robot IMU. To provide access to the GPS, an IGEPv2 board [44] is used to serve as a bridge between the GPS and the LEGO robot, it also serves as a data-logger to store the GPS sensor information along with the internal variables of the NXT robot. An USB hub connects the IGEP to the NXT with the setup (GPS-IGEP-HUB) being powered by a LiPo battery using a DC-DC converter to step-down the voltage to 5V. The fusion algorithm (Algorithm 3) is executed inside the NXT (not in the IGEPv2) to prove its applicability in limited resources platforms. The access to *L_GPS_* is handled using logic similar to the differential case, being the IGEPv2 the equivalent to the C.S. and S.S. in the outdoor framework. Also, the delay time Δ*T* is *measured* and used to adjust the *L_GPS_* used by the NXT.

The tests performed for both platforms are exposed next.

## 4. Experimental Results

To observe the performance of the proposed sensor fusion framework with the event based approach, in this section, several tests are performed in the platforms for indoors and outdoors scenarios. Also, an execution time test of the proposed algorithms is presented.

### 4.1. Local Fusion Performance Tests

**Figure 5.**
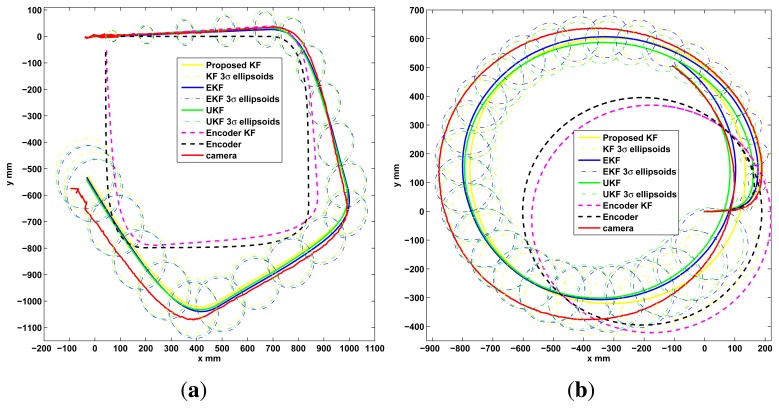
Algorithms performance in the differential platform, simulation test. (**a**) Square; (**b**) Circle.

**Figure 6.**
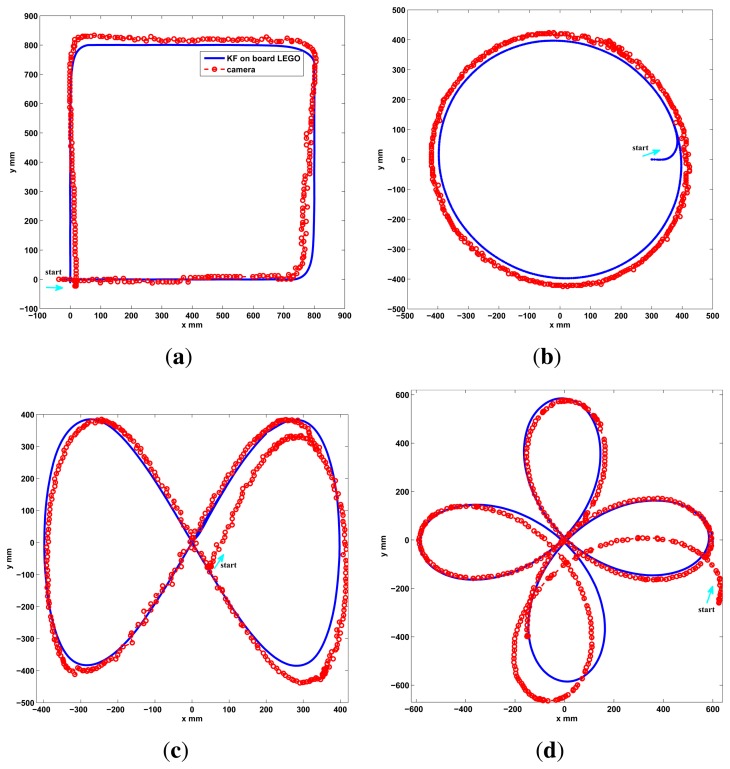
Implemented test, KF onboard the differential LEGO NXT. (**a**) Square; (**b**) Circle; (**c**) Lemniscate; (**d**) Polar Rose.

To observe the correctness of the local fusion execution, the first test is done without the event based global correction *EBGC* or any global information (*L_GM_*). The robot is set to follow a square and a circular reference trajectory using only the encoder measurements while recording the local sensor data and the C.S. recorded information. This data is employed in an Matlab® simulation to compare the proposed KF (Algorithm 4) with an EKF (Algorithm 1), an UKF ([5,45], same (*x, z, u*)*_k_* as Algorithm 1), and a KF version with *z_k_*[*υ_enc_ ω_enc_*]*^T^* (encoder KF) along with Equation (3) as the estimation model to show the performance of the KF alone, without using the proposed sensor fusion with the dynamic model. This is shown in Figure 5, where the proposed algorithm show a good performance. From this figure it is clear that the KF, EKF and UKF filters perform very similar among them when using the sensor fusion with the proposed model and no global information. But in the case of the KF (Algorithm 2) it uses less computational resources. Also, using the encoders only or the encoder KF is insufficient for this platform. In the second test, the performance of Algorithm 2 implemented on the differential LEGO NXT is shown in the experimental results of Figure 6. Again good pose estimation is observed as the robot follows different desired trajectories.

### 4.2. Event Based Algorithm Parameters

**Figure 7.**
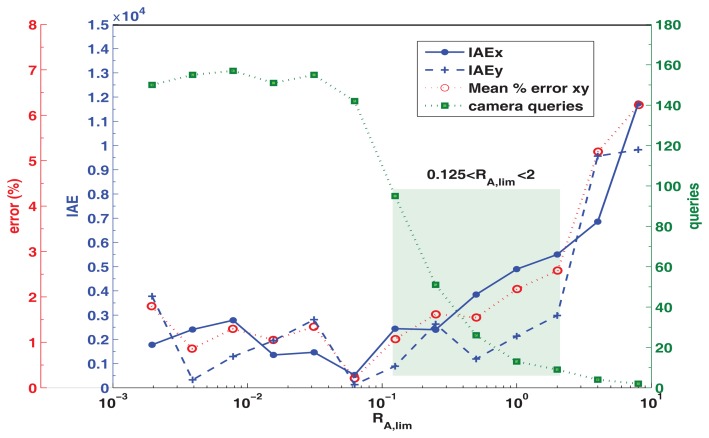
Relation between *R_A,lim_*, IAE performance, mean percentage error and camera queries for a 3 min square trajectory test, differential robot.

The parameters of the event based approach are analyzed in the following tests. The relation between *R_A,lim_*, the performance index *IAE* (integral of the absolute error) and the number of camera queries is shown in Figure 7 for a 3 min test of the differential robot following a square trajectory. From this test, it is clear that the performance index IAE (in the position *x*,*y*) increases with *R_A,lim_*, this is expected because a greater event level will produce less global updates, generating an increase in the pose estimation error and thus in the IAE. On the other hand, a decrease in the *R_A,lim_*, values will improve the performance, producing a lower value of the IAE indicator down to a limit, defined by the maximum number of calls that the platform can made (according to the available resources and the communications delay Δ*T*). From this figure it can also be observed that there is a tradeoff between the number of calls and the performance IAE ruled by the used *R_A,lim_*, value. Less resources and global sensor queries are obtained with *R_A,lim_*, > 2 but it will also produce a greater IAE. On the contrary, values of *R_A,lim_*, < 0.125 generate smaller IAE values but with more resources and camera queries used. With this, the recommended range of the event level parameter is 0.125 < *R_A,lim_* < 2, as these values will give a solution of compromise, with an adequate IAE and fewer camera calls.

In this Figure 7 an additional axis is added to show the mean *x*, *y* equivalent percentage error, obtained for each individual test by measuring the corresponding IAE of a one percent upscale in the *x* or *y* trajectory, and using this value to scale the corresponding IAE values from the tests. For the recommended *R_A,lim_* range, an error increase from 1.1% to 2.6% is expected (from 95 to 9 calls in 3 min), if *R_A,lim_* > 2 then the error can increase up to 6.2% (2 calls in *3* min) and if *R_A,lim_* < 0.125 then a minimum error of 0.2% can be obtained but with great resources and communications cost (142 calls or more in 3 min). From this it is clear that the proposed event based algorithm with and adequate *R_A,lim_* can obtain an acceptable IAE and percentage error while saving computational and communication resources by performing just the necessary queries to the global sensor to update the local estimation. This analysis can be performed for different platforms in order to obtain the optimum *R_A,lim_* range according to the setup employed.

**Figure 8.**
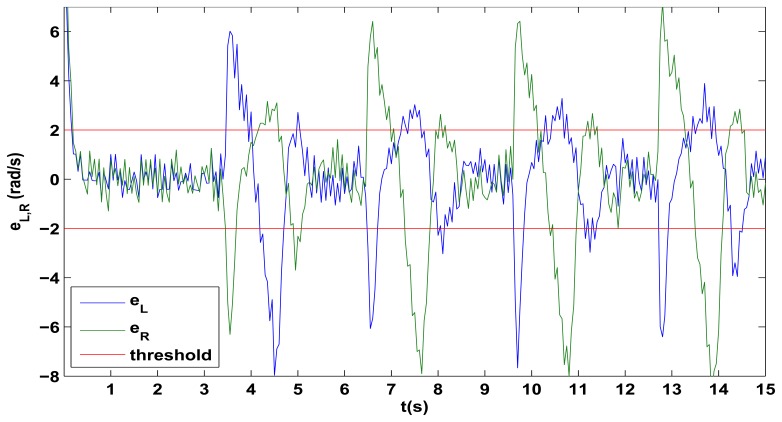
Reference error evolution in the PID control of the Left and Right wheels velocities *e_L,R_* and threshold definition.

The hard motion detection can be implemented in several ways according to the physical characteristics of the platform and the available sensors. For the proposed platforms, a hard turn or movement can be detected by observing the errors *e_L,R_* between the robot wheels velocities and the desired references, as this error increases when the direction changes suddenly and then rapidly decreases due the actuation of the PID controllers. The evolution of *e_L,R_* is observed in Figure 8 for the initial part of the square trajectory test, from it, a dual threshold can be established to define whether the robot is turning or not. By this, if |*e_L,R_*| > 2 then the robot is considered to be in a hard motion and thus, no global update must be performed. Finally, to allow the stabilization of the robot and to avoid the update in the case there are two consecutive hard movements, the algorithm can wait a predefined time (when returning to |*e_L,R_*| < 2) and then try to perform the global update. This waiting time depends on the settling time of the PID control of the wheels velocities, which in case of the proposed Lego platform, a minimum of 1s should be used due the noisy characteristic of the *e_L,R_* errors evolution. In case of other platforms, the *e_L,R_* errors evolution must be studied in order to obtain the error threshold |*e_L,R_*| and the corresponding waiting time.

### 4.3. Event Based Algorithm, Indoor Tests

**Figure 9.**
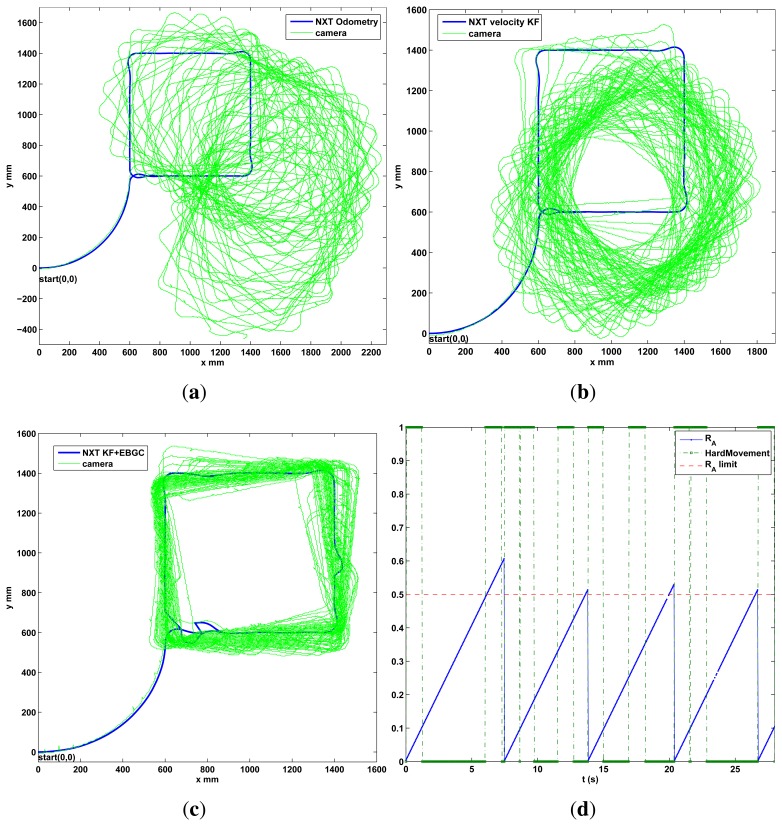
30 min run, methods comparison, differential robot. (**a**) Odometry from encoders; (**b**) Algorithm 2 without *L_GM_*; (**c**) Algorithm 2 with *L_GM_* and EBGC, *R_A,lim_* = 0.5; (**d**) *R_A_* evolution.

The performance of the event based fusion framework with Algorithm 2 is observed in a long distance run. The robot is set to follow a square trajectory for 30min, as shows the experimental results of Figure 9. In this, the robot using encoder-odometry only works well in the first lap but the estimated pose quickly diverges from the actual one. The proposed KF in Algorithm 2 *without* global update works well at first, but the trajectory diverge slowly as the error covariance increases. But in the case of the KF, Algorithm 2, with the event based update using *L_GM_*, the pose is estimated properly as the trajectory remains very similar to the square reference and the pose covariance does not increase indefinitely (estimation error is bounded). The evolution of *R_A_* is shown in Figure 9d for the first seconds of the test in Figure 9c. It shows how the global update is requested when *R_A_* exceeds a *R_A,lim_* = 0.5, for example, at time 6.25 s. At this moment, the robot checks the hard motion indicator. As it is “true” the camera was only requested and the pose was not updated. The algorithm waits 1s and at time 7.25 s it checks again. As the indicator is “false”, in this case the condition is cleared so the camera is requested again and the pose is updated at second 7.45 with a reset in the error covariance.

The IAE performance indicator has been obtained for the *x* and *y* position coordinates for the Figure 9 as it can be used as the main comparison of the proposed event based algorithm with the traditional odometry and the local fusion method. The results are summarized in the Table 2 from which the performance improvement obtained with the event based approach can be observed more clearly.

**Figure 10.**
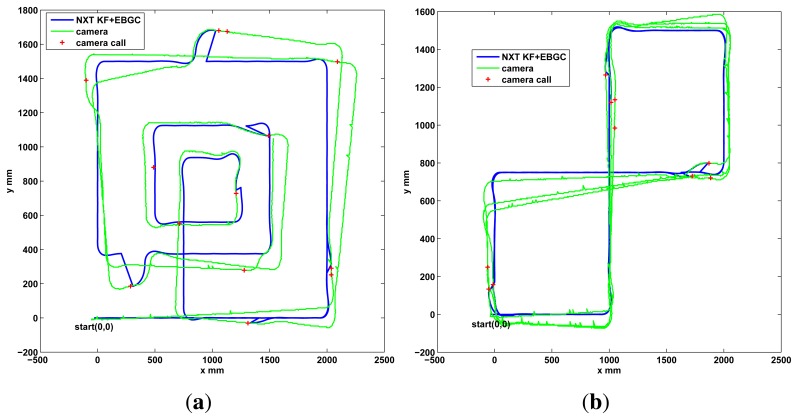
Five minutes run, global update performance, differential robot. (**a**) Square Spiral; (**b**) Double square.

As a final performance test for the differential robot, two new trajectories are shown in the experimental results of Figure 10. Again a good performance is observed, with Algorithm 2, since the robot follows the desired path and the estimation does not diverge due the event based approach. A video of the tests performed can be found in http://wks.gii.upv.es/cobami/webfm_send/5.

**Figure 11.**
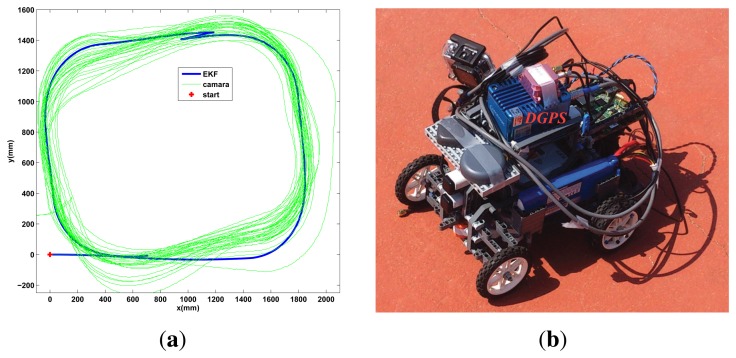
Ackermann indoor test with the zenithal camera and outdoor setup with DGPS. (**a**) indoor test, 30 min; (**b**) Outdoor setup with DGPS for validation.

Prior the outdoor test, the Ackermann robot is tested indoors in a long distance run using the camera setup instead of the GPS. The robot is set to follow a square trajectory for 30 min, as shows the experimental results of Figure 11a. Good performance is observed, with Algorithm 3, with the robot following the desired path and without divergence in the estimation due the event based approach.

### 4.4. Event Based Algorithm, Outdoor Tests

**Figure 12.**
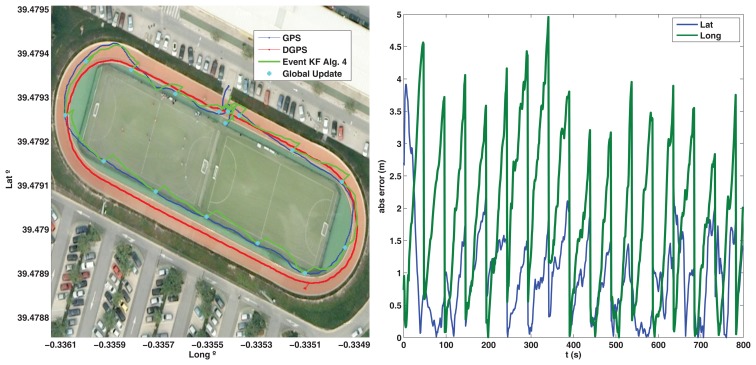
Ackermann Outdoor test, validation with DGPS.

The outdoor test is performed for the Ackermann robot, a long distance run is done following an outdoor velodrome track in a clockwise sense. A differential GPS (DGPS) is added to the platform (Figure 11b) to validate the result and obtain a more precise measurement of the real trajectory followed, but its information is not used by the robot to estimate the pose (only the IG-500 GPS is used in the event based scheme). The trajectory followed and the absolute estimation error is shown in Figure 12. Good performance is observed with Algorithm 3, since the estimation does not diverge due the event based approach (the estimation error is bounded) and the estimated pose is similar to the DGPS measurement. By modifying *R_A,lim_* the accuracy can be adjusted to allow the robot more queries to the GPS depending on the resources available, but the overall accuracy will depend on the GPS sensor used.

The execution time of the different algorithms is analyzed next.

### 4.5. Run Time test

**Figure 13.**
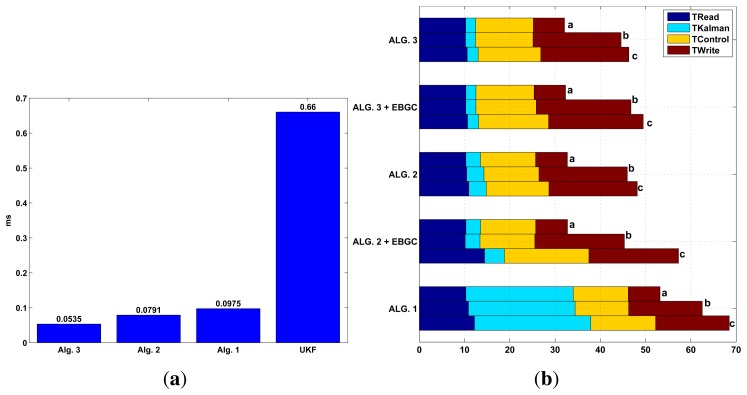
Run Time tests. (**a**) Mean Execution time test, PC simulation; (**b**) Execution time test, experimental results onboard the LEGO NXT.

To evaluate the run time efficiency of the proposed algorithms, a simulation of several KF algorithms using Matlab® running on a computer (2.4 GHz with 4 GB RAM) is performed. This is shown in Figure 13a where the first two columns correspond to the proposed methods KF in Algorithm 2, and EKF in Algorithm 4 that are compared to Algorithm 1 and an UKF (same (*x*, *z*, *u*)*_k_* as Algorithm 1). From this test, it is clear that the proposed KF Algorithm 2 and EKF Algorithm 4 have less computational effort (as they uses less *ms* of processor time) to obtain the pose estimate when comparing to the traditional approach Algorithm 1 or when comparing to the newer approach of the UKF. Although Algorithm 2 uses less time, it has similar performance as the more complex ones, as it was shown in the Figure 5.

A second test, where the execution time is measured for the different tasks running inside the robot, is performed. In this, the proposed KF Algorithm 2 and EKF Algorithm 4 are tested (with and without the event based approach) and compared with the time based EKF Algorithm 1. The task measured is the sensor reading (with calibration and preprocessing), the Kalman fusion algorithm, the control algorithm (navigation and motor control) and the write task which store the variables needed for supervision in a text file. This is shown in Figure 13b. Execution times are measured every cycle over a one minute test; and, as they are not constant, three cases are presented. Case *a* shows the mean task time per cycle, case *b* shows the time of the worst execution, and case *c* shows all the maximum times measured during the test although they don't occur at the same time at any cycle (or through the 30 min run), but is an indicator of worst case possible. This test show that only the proposed methods fulfill the sample time of 50 *ms* for cases *a* and *b*, being the KF Algorithm 2 the less resource consuming of all filters. The proposed EKF exceed the *T_s_* only in case *c*, but this is only a theoretical case, in all the tests performed this case never showed. As for the Algorithm 1, this exceeds *T_s_* in all the cases, not being suitable for being used in this robot where this sample time is needed. With this tests and the one in Figure 13a, the UKF will also exceed the required *T_s_*. Also, the memory limitations of the robot will not allow the implementation of any other task when using the Algorithm 1 or the UKF if *T_s_* = 50 *ms* and when the calculation of *K_k_* is performed in every cycle.

## 5. Conclusions

An efficient sensor fusion framework using the event based KF with a particle dynamical model of a differential wheel mobile robot for indoors navigation and an Ackermann mobile robot for outdoor navigation, have been presented as a solution for the localization problem. The results show that the performance of the proposed sensor fusion framework is similar to those obtained by using more complex EKF and UKF strategies with time based update and larger state and measurement vectors, but with a faster execution and less memory usage, allowing the implementation of the algorithm inside a limited resource robot while leaving enough resources for other tasks that must be executed inside the robot.

The sensor fusion framework can be adapted to other platforms by adding more or less sensors according to the robot capabilities, adjusting the KF matrices and vectors as needed. If the robot has very limited resources, this method can work using only the encoder measurements and one heading angle/rate sensor. Also, both accelerometers can be substituted for a model (identified from experimental data) that relates the motors control action and the linear accelerations of the wheels, to use as inputs in the proposed dynamic model. On the other hand, in an advanced robot with more resources, the event approach can be used to save bandwidth (reducing the number of queries to a global sensor) while substituting the KF with an UKF to improve accuracy.

Due to the evolution of the error covariance observed in the results, the event based method is necessary to correct the estimated pose. The results show an improved estimation and performance over the traditional odometry or with a fusion method without global sensor. Also, the event based approach reduces the processor usage, communications and battery consumption in the mobile robot, as it updates the pose only when it is necessary.

As future work, the method can be extended into different mobile robots configurations such as the Omnidirectional by adding particles to the proposed equivalent dynamical model. These particle-equivalent models can also be compared with more traditional dynamic and kinematic models in terms of accuracy, computational performance and implementation feasibility. Additionally, different sources of global information can be used, for example a scanning laser rangefinder to extend the method into a SLAM algorithm. Finally the event approach can be extended to provide a solution to the multirobot cooperative localization problem.

## Figures and Tables

**Table 1.** Dynamically equivalent particle system parameters.

**Robot Shape**	**Inertia Moment**	*&gamma*	***R****_N_*	**λ**
Thin Ring equivalent to (3) 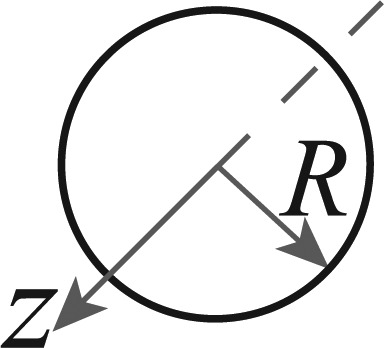	*I_G_* =*M_G_ R*^2^	0.5	*R*	$\frac{1}{R}$
Solid Cylinder 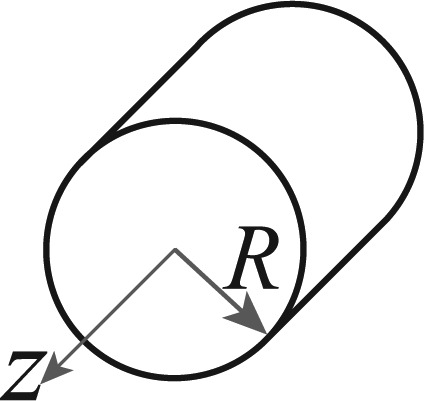	$I_{G}=\frac{M_{G}R^{2}}{2}$	0.5	$R\sqrt{0.5}$	$\frac{1}{R\sqrt{0.5}}$
Solid Box 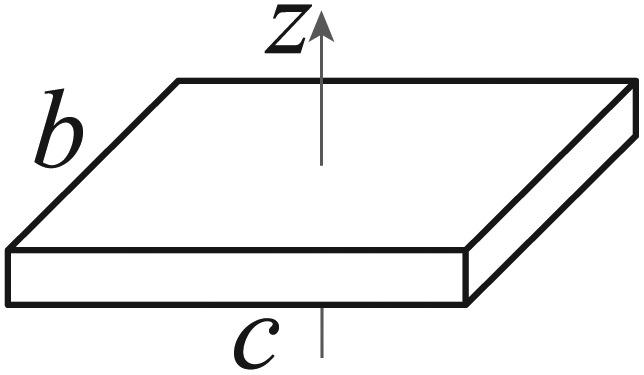	$\begin{array}{c} I_{G}=\beta M_{G}\\ \beta =\frac{1}{12}(b^{2}+c^{2}) \end{array}$	0.5	$\sqrt{\beta }$	$\frac{\sqrt{\beta }}{\beta }$

**Table 2.** IAE performance and percentage errors for the 30 min run, differential robot.

**Test in** figure 9	**IAE**			**Error(%**)	**Improvement over odometry(%)**

	*x*	*y*	**mean** *xy*	**mean** *xy*	**mean** *xy*
Odometry	9.541×10^5^	6.750 *×* 10^5^	8.146 *×* 10^5^	46.057	–
*Algorithm 2 without L_GM_*	2.949 *×* 10^5^	4.376 *×* 10^5^	3.663 *×* 10^5^	20.734	238.9
*Algorithm 2 with L_GM_*	6.221 *×* 10^4^	5.342 *×* 10^4^	5.781 *×* 10^4^	3.270	1398.8

## A. Appendix

### Dynamically Equivalent Particle System

To study the robot rigid body as a dynamically equivalent particle system, formed by two particles of mass *M_L_* and *M_R_* joined by a massless connector of constant length *b* (Figure 1b), the following conditions must be met [37]:

- Mass Conservation: *M_G_* = *M_L_* + *M_R_*
- Mass Center Conservation: *M_L_ R_L_* = *M_R_ R_R_*
- Moment of Inertia Conservation: $M_{L}R_{L}^{2}+M_{R}R_{R}^{2}=I_{G}$

By defining *R_L_* = *R_R_* = *R_N_* the masses can be obtained as it is shown in Equation (15). The masses *M_L_* and *M_R_* are proportional to the total mass *M_G_* expressed by the constant *γ*. Also the constant λ is defined as the ratio between the mass and moment of inertia of the rigid body multiplied by the distance between the mass center and the particle.

(15) $$\begin{array}{l} M_{L}=M_{R}=\gamma M_{G}\\ R_{N}^{2}=\frac{I_{G}}{M_{G}}\\ \lambda =\frac{M_{G}R_{N}}{I_{G}} \end{array}$$

By substituting *I**_G_* in Equation (15), the parameters of the particle system are obtained for different robots shapes as shown in Table 1. To obtain the dynamic model, first, the second Newton's Law is applied to both the rigid body and the particle system shown in Figure 1b to obtain Equations (16a) and (16b).

(16a) $$\begin{array}{l} \sum F=F_{R,g}+F_{L,g}=M_{G}a\\ \sum \tau =\tau_{R,g}-\tau_{L,g}=I_{G}\alpha \end{array}$$

(16b) $$\begin{array}{l} \sum F=F_{R,p}+F_{L,p}=M_{R}a_{R}+M_{L}a_{L}\\ \sum \tau =\tau_{R,p}-\tau_{L,p}=R_{N}(M_{R}a_{R}-M_{L}a_{L}) \end{array}$$

Both systems Equations (16a) and (16b) are dynamically equivalent when using Equation (15) and the parameters from the Table 1, but also the forces and torques applied to Equations (16a) and (16b) should be the same. The forces satisfy *F_R,g_* + *F_L,g_* = *F_R,p_* + *F_L,p_* as they are applied by the same motors. But in order to the torques satisfy *τ_R,g_*−*τ_L,g_* = *τ_R,p_* − *τ_L,p_* the distances should meet *b* = 2*R_N_*. As *R_N_* is defined by Table 1 (is fixed by Equation (15), it cannot be modified), the distance between wheels in the robot should be adjusted. This can be done in the design stage or by using a configurable structure robot (for example the LEGO ®NXT that is used in the tests). If the relation *b* = 2*R_N_* does not hold for a particular robot, a similar model can be obtained using a three particle system (the same two particles as above plus one in the mass center) that will allow an arbitrary choice of *R_N_*. Setting *b* = 2*R_N_* in Equations (16a) and (16b) and solving for *a* and *α* by substituting Equation (15), the robot linear and local dynamic model is obtained in Equation (4).
